# Avoiding thermal injury during near-infrared photoimmunotherapy (NIR-PIT): the importance of NIR light power density

**DOI:** 10.18632/oncotarget.20179

**Published:** 2017-08-11

**Authors:** Shuhei Okuyama, Tadanobu Nagaya, Fusa Ogata, Yasuhiro Maruoka, Kazuhide Sato, Yuko Nakamura, Peter L. Choyke, Hisataka Kobayashi

**Affiliations:** ^1^ National Institutes of Health, National Cancer Institute, Molecular Imaging Program, Bethesda, Maryland, 20892, United States

**Keywords:** near-infrared photoimmunotherapy, thermal effect, thermal imaging, power density

## Abstract

Near-infrared photoimmunotherapy (NIR-PIT) is a newly-established cancer treatment which employs the combination of an antibody-photoabsorber conjugate (APC) and NIR light. When NIR light is absorbed by APC-bound tissues, a certain amount of heat is generated locally. For the most part this results in a subclinical rise in skin temperature, however, excessive light exposure may cause non-specific thermal damage. In this study, we investigated the potential for thermal damage caused by NIR-PIT by measuring surface temperature. Two sources of light, laser and light emitting diode (LED), were compared in a mouse tumor model. First, we found that the skin was heated rapidly by NIR light regardless of whether laser or LED light sources were used. Air cooling at the surface reduced the rise in temperature. There were no associations between the rise of skin temperature and tumor volume of the treated tumor, or APC concentration. Second, we investigated the extent of thermal damage to the skin at various light doses. We detected burn injuries 1 day after NIR-PIT, when the NIR light was at a power density higher than 600 mW/cm^2^. Successful treatments at lower power density could be achieved if the total light energy absorbed by the tumor was the same, i.e. by extending the duration of light exposure. In conclusion, this study demonstrates that thermal injury after NIR-PIT can be avoided by either employing a cooling system or by lowering the power density of the light source and prolonging the exposure time such that the total energy is constant. Thus, thermal damage is preventable side effect of NIR-PIT.

## INTRODUCTION

Near infrared photoimmunotherapy (NIR-PIT) is a newly-established cancer therapy utilizing an antibody-photoabsorber conjugate (APC), which binds to antigens expressed on the cellular membrane of cancer cells. The APC is subsequently activated by NIR light irradiation. Since the combination of the APC and NIR light rapidly induces necrotic/immunogenic cell death only in APC-bound cells, the treatment is highly selective for cancer [1, 2]. NIR-PIT has entered clinical testing using the anti epidermal growth factor receptor (EGFR) antibody, cetuximab, and the photoabsorber, IR700. This Phase 1 and 2 trial in patients with recurrent inoperable head and neck cancer of NIR-PIT was approved by the US Food and Drug Administration (FDA) in April 2015, and is currently open (https://clinicaltrials.gov/ct2/show/NCT02422979).

In NIR-PIT, appropriate dosing of NIR light is important, because excessive light exposure can result in thermal damage which is not necessary for the treatment to be effective. Although appropriate NIR dose varies with tumor cell type, size, and light source, etc., NIR-PIT proved effective between a light dose of 10–100 J/cm^2^ [3–5]. Interestingly, the most important determinant of NIR-PIT treatment success was that the tumor received at least a certain threshold of total light regardless of the power density or exposure time of the light source [6].

NIR light at wavelengths of approximately 700 nm exhibits relatively high optical penetration within living tissue because at this wavelength there are absorbance minima for endogenous chromophores (water, hemoglobin, collagen, etc.) [7]. Light energy absorbed by objects generally converts to thermal energy, resulting in increasing temperature. Therefore, excessive light exposure to biological tissue readily causes thermal side effects including burn injury. The risk of thermal damage increases at temperatures higher than 43°C [8]. Since the actual temperature shift has not been previously measured during NIR-PIT, we measured the temperature shift within tissues exposed to NIR during NIR-PIT in the skin of tumor-bearing athymic mice. The results should be helpful in optimizing NIR light delivery so as not to cause thermal discomfort or injury.

## RESULTS

### Air cooling can suppress temperature increase

**Figure 1:**
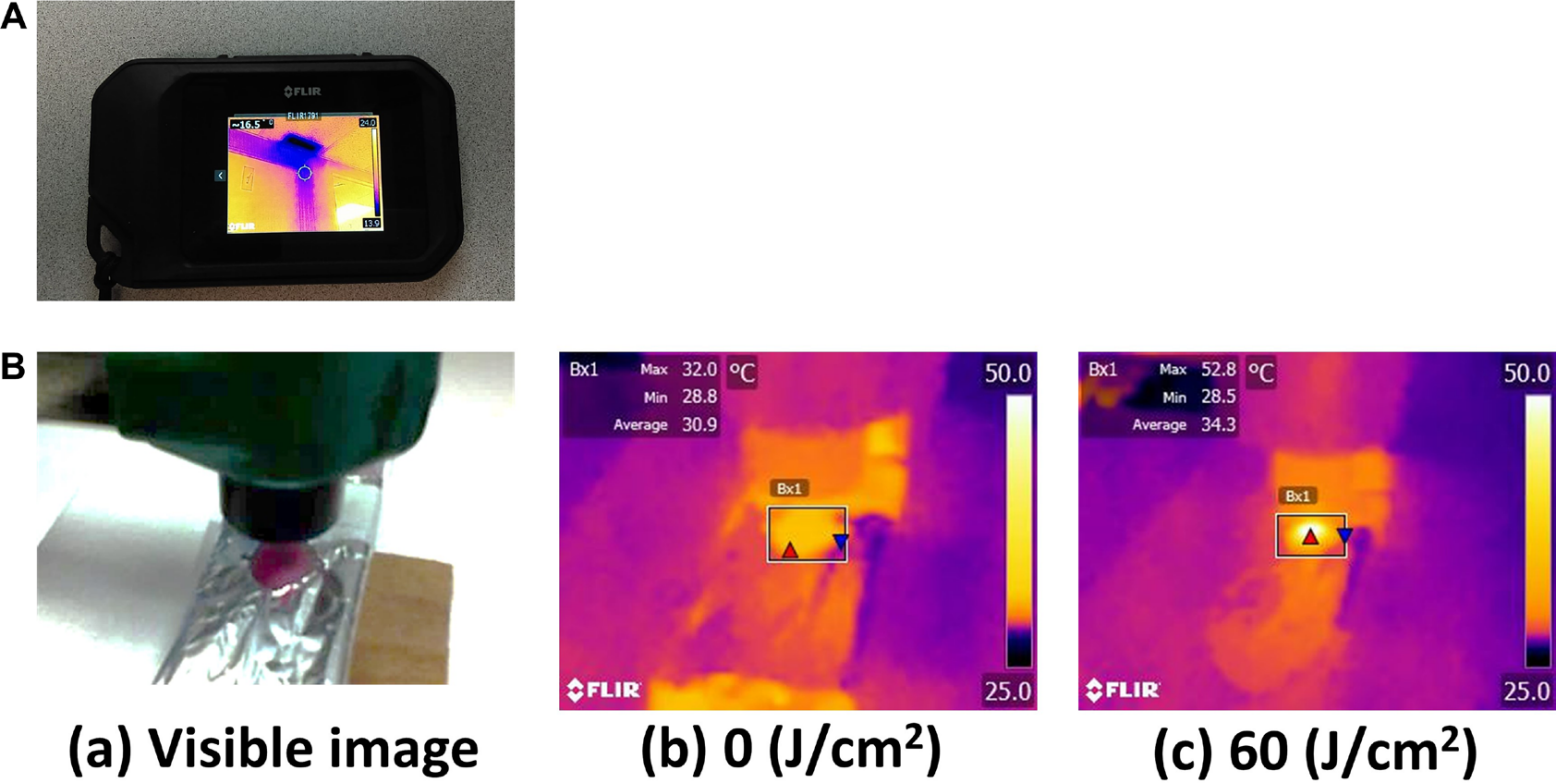
Overview of the thermal imaging camera and thermal image (**A**) The thermal imaging camera used in this study. (**B**) A visible image and the thermal images taken by the camera before and after NIR irradiation. The maximum temperature exposed to NIR light, was measured. The temperature of mouse skin increased with NIR light irradiation gradually.

**Figure 2:**
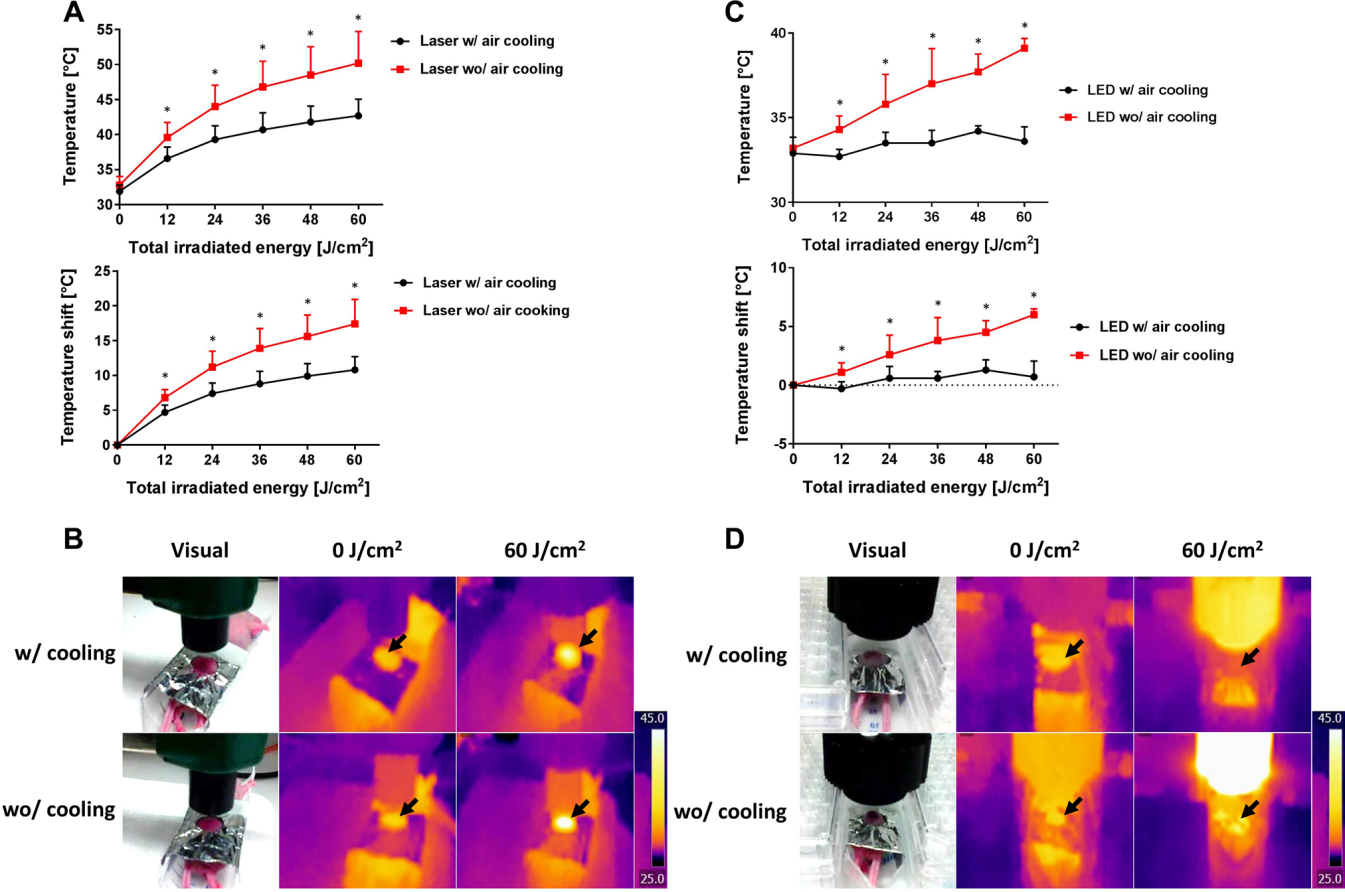
Temperature increases caused by laser or LED light, with or without air cooling (**A**) Temperature increases during laser exposure at a power density of 600 mW/cm^2^ with or without air cooling. The temperature increase could be ameliorated by about 40% using air cooling. (**B**) The visual images and thermal images during the laser exposure with or without air cooling. Black arrows indicate the light exposure areas. (**C**) Temperature increases with LED light exposure at the power density of 50 mW/cm^2^ with or without air cooling. Temperature changes were barely observed with air cooling. (**D**) The visual images and thermal images during the LED irradiation with or without air cooling. Black arrows indicate the light exposure areas. Data are means ± SEM. *n* = 5 in each group. ^*^P < 0.05 versus the other group.

In order to investigate the relationship between temperature changes at tissues and NIR light dose (whether from laser or light emitting diode (LED)), the temperature of mouse skin was measured during NIR-PIT via a thermal imaging system (Figure 1). The change in temperature was compared with or without air cooling using a desktop fan. Both NIR exposure with the laser and the LED caused the mouse skin to heat, although the effect was dependent on the light dose (Figure 2A, 2B). When the skin was exposed with 60 J/cm^2^ at 600 mW/cm^2^ using the laser, skin temperature reached 50.2 ± 4.5°C (Δt = 17.4 ± 3.5°C) without air cooling (*n* = 5). On the other hand, the same exposure dose with air cooling resulted in a maximal skin temperature of 42.7 ± 2.4°C (Δt = 10.8 ± 1.9°C) (*n* = 5). Air cooling decreased the temperature variation by about 40%. When the light source was switched to LED light at 60 J/cm^2^ using 50 mW/cm^2^ NIR light irradiation without air cooling resulted in mouse skin temperatures of 39.1 ± 0.6°C (Δt = 6.0 ± 0.5°C) (*n* = 5), whereas with air cooling the skin only reached 33.6 ± 0.9°C (Δt = 0.7 ± 1.4°C) (*n* = 5) (Figure 2C, 2D). In this case, the skin was minimally heated by administration of 60 J/cm^2^ LED light irradiation provided air cooling was available.

### The increase in skin temperature did not correlate with APC dose

**Figure 3:**
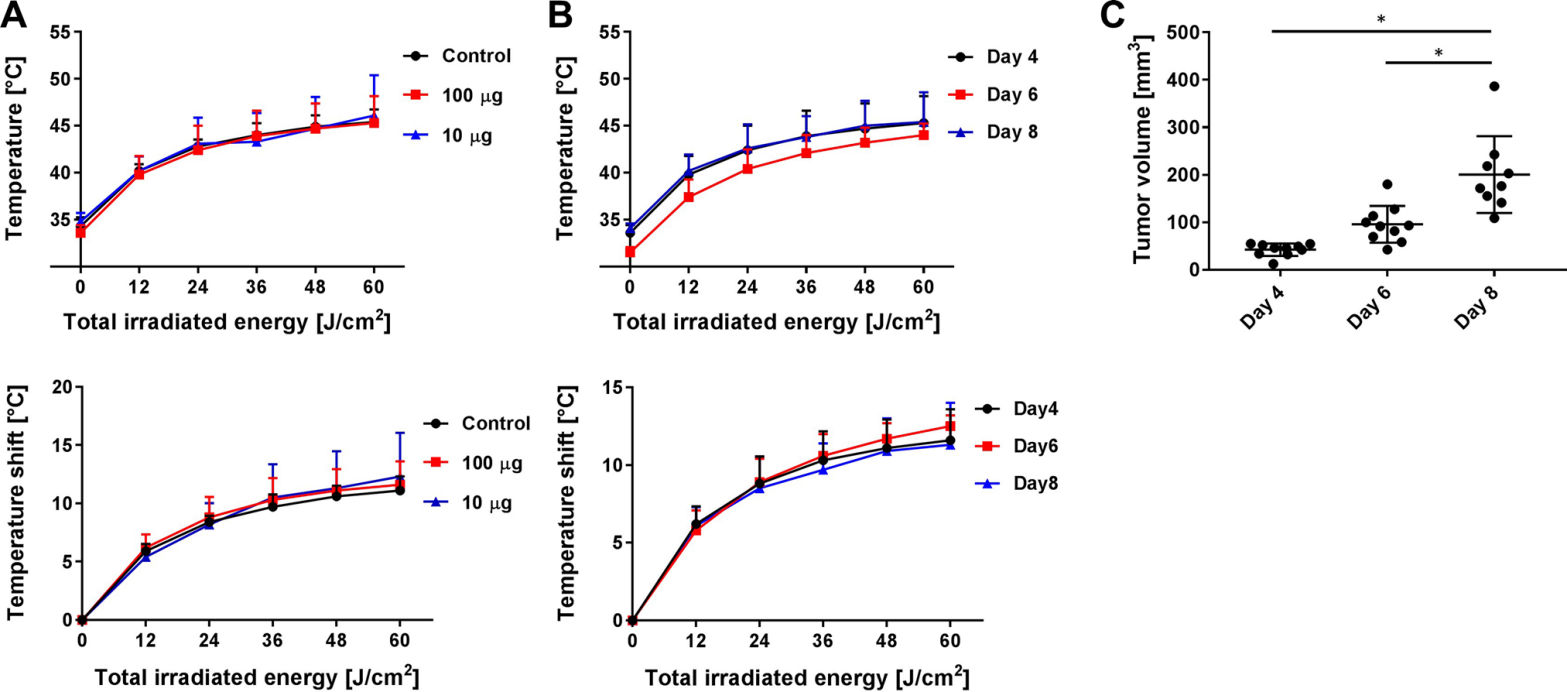
Temperature changes with APC dose and tumor volume (**A**) The effect of APC dose (0, 10 or 100 μg) on temperature changes at a power density of 600 mW/cm^2^ with air cooling. There were no significant differences among the tested groups. *n* = at least 5 in each group indicating little effect of APC dose. (**B, C**) Temperature increases at different tumor volumes (day 4, 6 and 8 cell incubation period) using laser irradiation at 600 mW/cm^2^ with air cooling, and tumor volume in each group. There were no significant differences among them. *n* = at least 5 in each group. Data are means ± SEM. ^*^P < 0.05 versus the other group. This indicates temperature changes are not dependent on tumor volume.

NIR light is absorbed by APC as well as chromophores, and thus, its energy will be converted to heat. Thus, tumors containing high concentration of APC could theoretically exhibit higher temperature increases than tumors with lower concentrations of APC. To elucidate whether APC concentration influences skin heating, we measured skin temperature during NIR-PIT at different volumes of APC administration (0, 10 or 100 μg). The skin was exposed with 60 J/cm^2^ at 600 mW/cm^2^ using the laser, reaching a temperature of 45.4 ± 1.3, 46.1 ± 4.3 and 45.3 ± 2.9°C at 0, 10 and 100 μg, respectively (Δt = 11.1 ± 1.2, 11.6 ± 2.0 and 12.3 ± 3.8°C, respectively) (*n* = at least 5 in each group) (Figure 3A). There were no significant differences among tested groups. Furthermore, we also measured skin temperature in tumors that were younger or older, because, in theory, as tumors grow, more antigens for binding the APC would become available. Each A431 tumor-bearing mouse at the day 4, 6 and 8 after xenograft was heated to 45.3 ± 2.9, 44.0 ± 1.2 and 45.4 ± 3.2°C, respectively (Δt = 11.6 ± 2.0, 12.5 ± 0.7 and 11.3 ± 2.7°C) (*n* = at least 5 in each day) (Figure 3B, 3C). There were no significant differences at different tumor sizes.

### Keeping skin temperatures lower than 50°C during NIR-PIT

**Figure 4:**
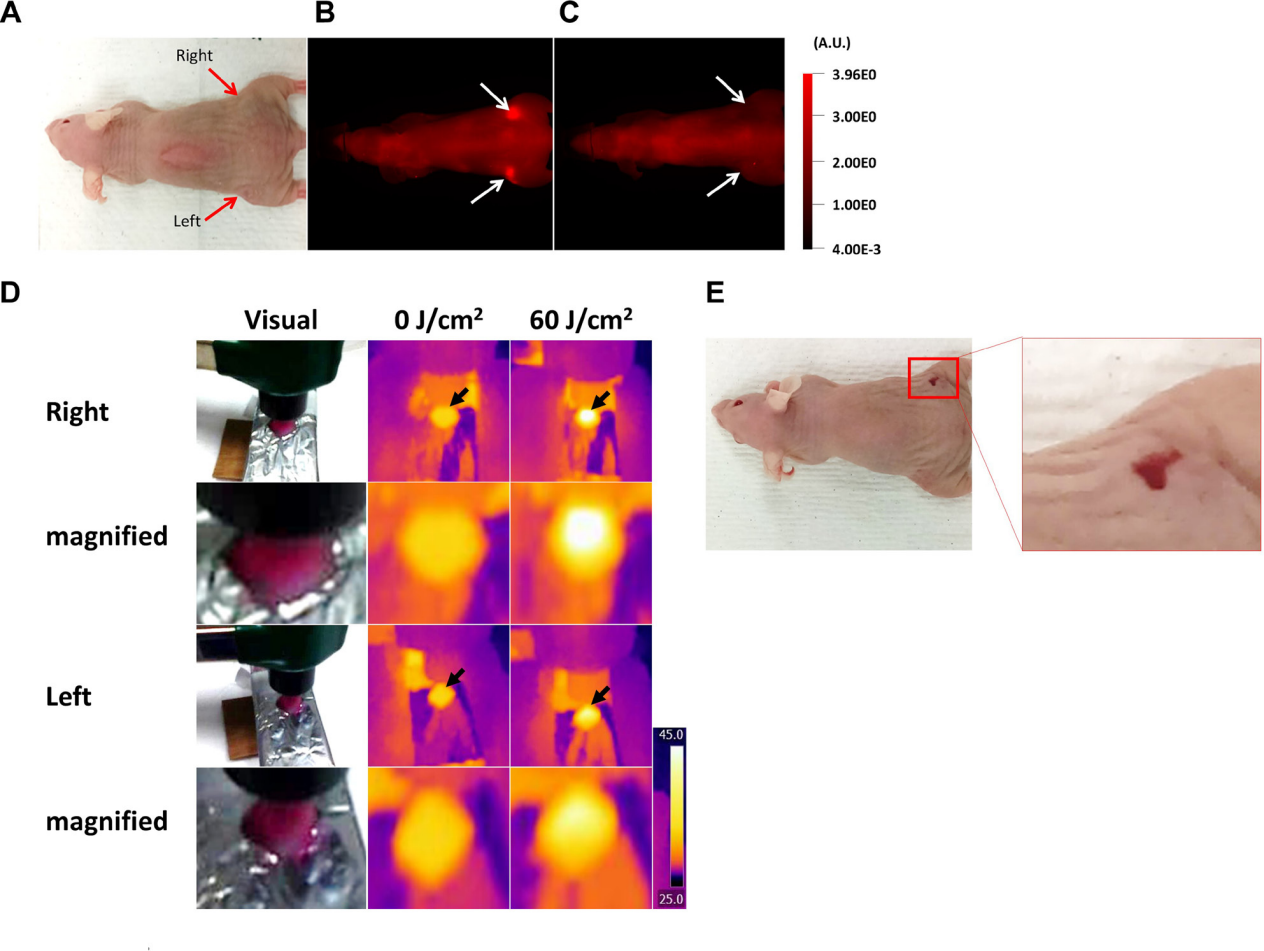
Thermal damage to mouse skin (**A**) Visual image before NIR-PIT. (**B, C**) IR700 fluorescence images before and after NIR-PIT. White arrows indicate the tumors. The fluorescence intensity of both tumors decreased after NIR light exposure. (**D**) The visual images and thermal images taken in the experiment above. Black arrows indicate the light exposure areas. The tumor at the right dorsum was heated to over 50°C at 60 J/cm^2^, whereas the left dorsal tumor was heated to under 50°C. (**E**) The visual image at 1 day after NIR-PIT. Only the tumor in the right dorsum exhibited a visual change suggestive of thermal injury.

During the relevant exposure times, visual damage to skin from thermal injury could be observed when it reached a temperature above 50°C. In this case, visible signs of erythema were present the next day (Figure 4). However, when the skin temperature was maintained below 50°C, no such damage was observed. Therefore, these results indicate that mouse skin might be damaged by NIR light irradiation when the temperature exceeded 50°C.

### Skin temperature increases are strongly dependent on the power density of NIR light

**Figure 5:**
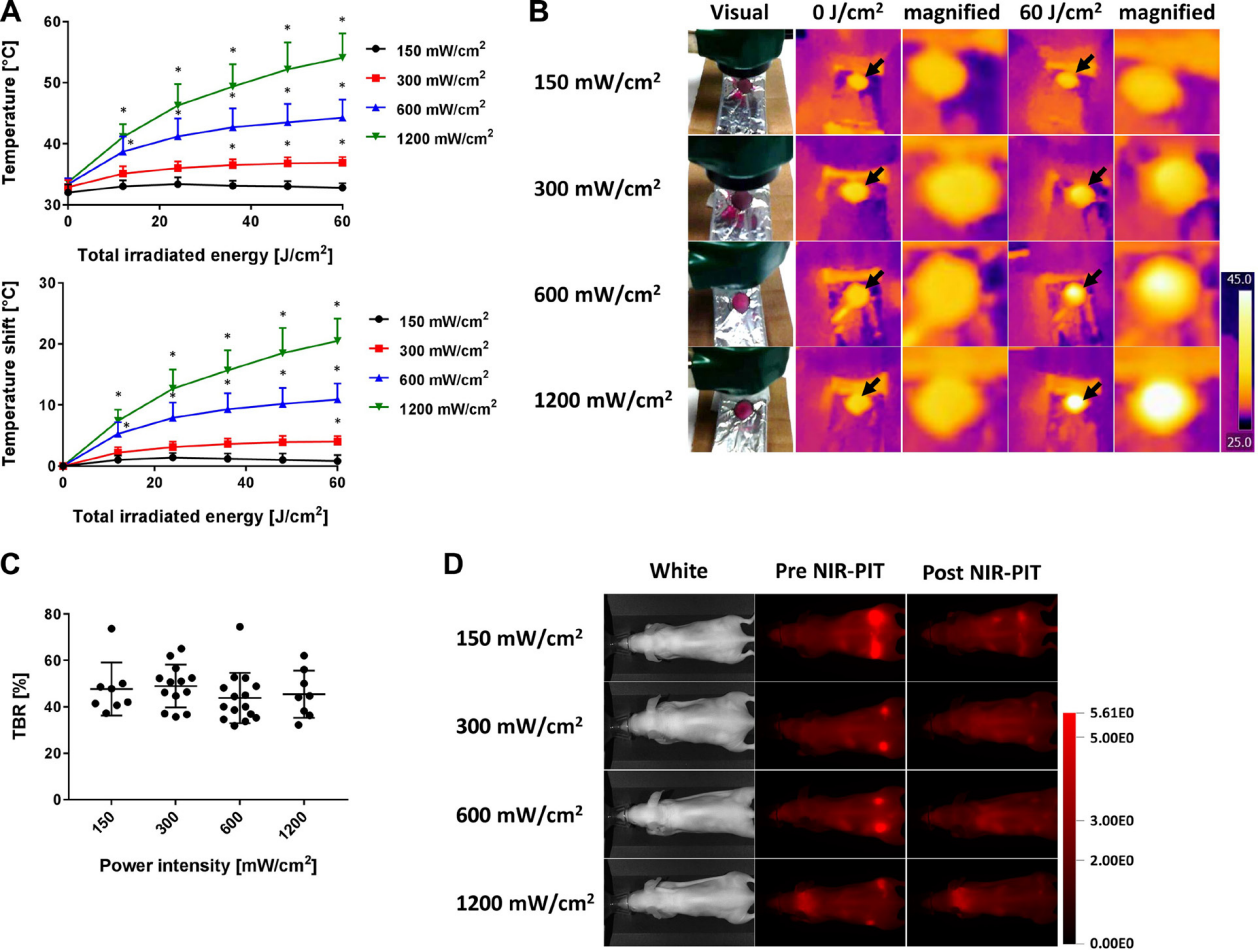
Temperature increases at several of power densities using laser light (**A**) Temperature increases after laser exposure of 60 J/cm^2^ at each of the following power densities (150, 300, 600 and 1,200 mW/cm^2^) with air cooling. The lower the power density, the lower was the temperature increase. (**B**) The visual images and thermal images after laser exposure with different power densities. Black arrows indicate the irradiated areas. At the 60 J/cm^2^, the higher the power density, the higher the temperature that was exhibited. (**C**) TBR at 60 J/cm^2^ in each group. No significant differences were seen among the groups. (**D**) Fluorescence images of IR700 before and after NIR-PIT at each power density. Data are means ± SEM. *n* = at least 8 in each group. ^*^P < 0.05 versus the 150 mW/cm^2^ group.

Temperature changes were measured during laser irradiation at different power density settings: 150, 300, 600 and 1,200 mW/cm^2^. The lower the power density, the lower was the rise in temperature of the mouse skin. When an exposure of 60 J/cm^2^ NIR light was delivered at different power densities, the skin temperatures during NIR-PIT increased as follows: 32.8 ± 0.8, 36.9 ± 1.0, 44.3 ± 3.0 and 54.1 ± 4.0°C (Δt = 0.8 ± 1.0, 4.0 ± 0.9, 10.9 ± 2.6 and 20.5 ± 3.7°C at 150, 300, 600 and 1,200 mW/cm^2^, respectively) (*n* = at least 8 in each group) (Figure 5A, 5B). There were no significant differences among each tumor-to-background ratio (TBR) (Figure 5C, 5D) (*P* > 0.05 at all combinations).

## DISCUSSION

A potential concern of administering NIR light is thermal injury which can occur if the power density of the light is too high. In NIR-PIT, when the NIR is applied, the light undergoes reflection, refraction, scattering, transmission and absorption by tissue. Only absorbed light contributes to heat generation in tissues. In general, excessive light exposure may cause over heating resulting in thermal injury. Therefore, caution must be exercised when applying NIR light to APC-bound tissue.

NIR-PIT can be performed with either laser or LED as the light source [4]. In this study, we demonstrated that the surface skin temperature increased depending on total light doses (Figure 2). Despite keeping the light dose constant, the observed temperature increase induced by the laser was greater than that induced by LED. This is most likely due to the inherent differences in power density between laser and LED light. The power density of the laser (600 mW/cm^2^) was substantially higher than the LED (50 mW/cm^2^). Thus, while the laser made the irradiation time shorter, it also led to increased light energy deposition in the exposed area in a short time, resulting in such high skin temperature. Increased air circulation provided by a simple house fan was effective for cooling down the skin sufficiently to avoid damage. In this study, we used the light source and the air fan separately. Integration of cooling fans during NIR-PIT could be useful in some cases.

NIR light is absorbed by not only chromophores such as water and hemoglobin, but also by IR700. However, we demonstrated that there was no significant difference in the skin temperature when either the volume of APC or the tumor size were different (Figure 3A–3C). This indicates that volume of APC and tumor size play a negligible role in the risk of thermal injury. However, in general, the additional heat contributed by the amount of APC in tumors was minimal compared with natural chromophores in the body.

While performing these experiments, we occasionally found thermal injury the day after NIR light irradiation (Figure 4). Such legions, which were shown in red color as typical acute thermal damages that were different from NIR-PIT cell killing effects which were shown in white, corresponded to locations that reached over 50°C, whereas no skin changes were never seen at lower temperatures. Therefore, to avoid excessive temperature increases at the skin, NIR light should delivered at 600 mW/cm^2^ or lower power density. We have previously reported that the efficacy of NIR-PIT is highly dependent on the NIR light dose, not its power density [6]. Although irradiation at lower light densities requires much more time to give the same light dose to target tissues, temperature increases were dramatically suppressed (Figure 5). In the clinical trial of NIR-PIT, NIR light is irradiated at a power density of up to 150 mW/cm^2^. Such a low power density, four times lower than the power densities associated with thermal injury, greatly lowers the risk of thermal damage. In addition, this problem has not been reported by conventional photodynamic therapy because phototoxicity of conventional photosensitizers to normal tissue is induced with much low energy of light before causing this thermal injury.

Recently, new type of cancer photodynamic therapies using genetically transfected photo-toxic fluorescent proteins were reported. Cancer cells expressing such fluorescent proteins were successfully treated with exposure of UVC [9–12]. The agents in the nucleus and/or the cytoplasm react with the light and results in the apoptotic cell death due to oxidation reaction. In contrast, NIR-PIT agents on the cell membrane react with the light and result rapid necrotic cell death due to photo-chemical reaction [2, 9, 10].

In this study, we demonstrate that excessive NIR light irradiation to mouse skin during NIR-PIT induces temperature increases that can cause thermal skin injury, especially when heated over 50°C. One approach to avoiding excessive heat by NIR irradiation is to simply provide increased air cooling to the target tissue. NIR-PIT can be performed safely with NIR light exposure at power densities at 600 mW/cm^2^ or lower without causing thermal injury.

## MATERIALS AND METHODS

### Reagents

Water soluble, silica-phthalocyanine derivative, IRDye700DX NHS ester (IR700; C_74_H_96_N_12_Na_4_O_27_S_6_Si_3_, molecular weight of 1954.22) was obtained from LI-COR Bioscience (Lincoln, NE). Panitumumab, a fully humanized IgG2 monoclonal antibodies (mAb) directed against EGFR, was purchased from Amgen (Thousand Oaks, CA). All other chemicals were of reagent grade.

### Synthesis of IR700-conjugated panitumumab

Conjugation of dyes with mAb has been previously described [13]. Briefly, panitumumab (1 mg, 6.8 nmol) was incubated with IR700 (66.8 μg, 34.2 nmol, 5 mmol/l in DMSO) in 0.1 mol/l Na_2_HPO_4_ (pH 8.5) at room temperature for 1 h. Subsequently, the mixture was purified with a Sephadex G25 column (PD-10; GE Healthcare, Piscataway, NJ). The protein concentration was determined with a Coomassie Plus protein assay kit (Pierce Biotechnology, Rockford, IL) by measuring the absorption at 595 nm (8453 Value System; Agilent Technologies, Santa Clara, CA). The concentration of IR700 was measured by its absorption to confirm the number of fluorophore molecules conjugated to each mAb. We abbreviate IR700-conjugated panitumumab as Pan-IR700.

### Cell lines and culture

A431 cells expressing EGFR were grown in RPMI1640 supplemented with 10% FBS and 1% penicillin-streptomycin in tissue culture flasks in a humidified incubator at 37°C in an atmosphere of 95% air and 5% carbon dioxide.

### Animal and tumor models

All *in vivo* procedures were conducted in compliance with the Guide for the Care and Use of Laboratory Animal Resources (1996), US National Research Council, and approved by the local Animal Care and Use Committee. Six- to eight-week-old female homozygote athymic nude mice were purchased from Charles River (Frederick, MD). During the procedure, mice were anesthetized with isoflurane. In order to determine tumor volume, the greatest longitudinal diameter (length) and the greatest transverse diameter (width) were measured with a caliper. Tumor volumes were based on caliper measurements and were calculated using the following formula for a prolate ellipsoid; tumor volume = length × width^2^ × 0.5.

### *In vivo* NIR-PIT

In order to compare thermal effects a 690-nm laser (BWF5-690-8-600-0.37; B&W TEK INC., Newark, DE) and a 690-nm LED bulb, (L690-66–60; Marubeni America Co., Santa Clara, CA) were used for NIR-PIT. Light was applied with or without air cooling using a desk fan (Holmes, Boca Raton, FL). A431 cells (2 million in phosphate-buffered saline) were injected subcutaneously in both the right and left dorsa of mice. Pan-IR700 (100 μg) was injected intravenously 5 days after injection of tumor cells. The mice were then randomized into 4 groups of at least 5 animals per group for the following treatments: (1) laser NIR light exposure with air cooling; (2) laser NIR light exposure without air cooling; (3) LED NIR light exposure with air cooling; (4) LED NIR light exposure without air cooling. Every NIR-light exposure was performed followed the Pan-IR700 injection by one day and were performed at a power density of 600 mW/cm^2^ with the laser and 50 mW/cm^2^ with the LED, for a total dose of 60 J/cm^2^.

For investigating the relationship between thermal effect and APC concentration, A431-tumor bearing mice at 5 days after tumor xenografting were administered Pan-IR700 as follows: (1) no agent (control); (2) 10 μg Pan-IR700; (3) 10 μg Pan-IR700. Each group included at least 5 mice. One day after Pan-IR700 administration, a laser light was applied at a power density of 600 mW/cm^2^ and total dose of 60 J/cm^2^ with air cooling.

For investigating the relationship between thermal effect and tumor volume, A431-bearing mice were administered 100 μg Pan-IR700 at 3, 5 or 7 days after tumor implantation. Each group included at least 5 mice. Each mouse received Pan-IR700 followed on the following day by NIR-light exposure at a power density of 600 mW/cm^2^ and total light dose of 60 J/cm^2^ with air cooling.

To compare mouse skin temperature during NIR light exposure with a variety of power density settings, A431-tumor bearing mice were administered Pan-IR700 (100 μg) intravenously. The mice were divided into 4 groups randomly, and NIR light irradiation via the laser was performed at the following power densities: (1) 150 mW/cm^2^; (2) 300 mW/cm^2^; (3) 600 mW/cm^2^; (4) 1,200 mW/cm^2^. Each NIR light dose was designed to reach a maximum light exposure dose of 60 J/cm^2^ with air cooling.

### Fluorescence imaging studies

Fluorescence images were obtained with a Pearl Imager (LI-COR Biosciences, Lincoln, NE) using a 700-nm fluorescence channel 24 h after intravenous injection of Pan-IR700, and immediately after NIR-PIT. For analyzing fluorescence intensities, regions of interest (ROIs) were placed on the tumor and the area adjacent to the tumor, then the average fluorescence intensities of each ROI were measured by Pearl Cam Software (LI-COR Biosciences, Lincoln, NE). The tumor-to-background ratio (TBR) of fluorescence intensities before and after NIR-PIT were calculated according to the following equation:

TBR = (F_A TUMOR_ - F_A BG_) / (F_B TUMOR_ - F_B BG_) × 100.

Where a F_A TUMOR_ is the fluorescence intensity of tumor after NIR-PIT, a F_A BG_ is the intensity of the background after NIR-PIT, a F_B TUMOR_ is the intensity of tumor before NIR-PIT and a F_B BG_ is the intensity of the background before NIR-PIT, respectively.

### Thermal imaging analysis

While each mouse was exposed to NIR light, both visible and thermal images were taken via a thermal imaging system (FLIR Systems, Inc. Wilsonville, OR) at about 20 cm distance. Total light dose varied from 0, 12, 24, 36, 48 to 60 J/cm^2^. To analyze temperatures, a single ROI was placed on each irradiated area, and then the maximum temperature in the ROI was calculated by FLIR tools (http://www.flir.com/instruments/display/?id=55473). The temperature variations were calculated by subtracting the temperature at 0 J/cm^2^ from each higher temperature, represented as Δt.

### Statistical analysis

Statistical analyses were carried out using GraphPad Prism version 7 (GraphPad Prism; GraphPad Software Inc., La Jolla, CA). For multiple comparisons, a one-way analysis of variance (ANOVA) followed by the Bonferroni's multiple comparisons test was used. Student's *t* test was also used to compare the temperature increase with or without air cooling. *P*-values of less than 0.05 were considered statistically significant.

## Abbreviations

PIT: Photoimmunotherapy
NIR: Near infrared
APC: Antibody-photoabsorber conjugate
mAb: Monoclonal antibody
EGFR: Epidermal growth factor receptor
FDA: Food and Drug Administration
ROI: Region of interest
LED: Light emitting diode
TBR: Tumor-to-background ratio
DMSO: Dimethyl sulfoxide
ANOVA: one-way analysis of variance.
